# Prospects for the Mechanism of *Spiroplasma* Swimming

**DOI:** 10.3389/fmicb.2021.706426

**Published:** 2021-08-27

**Authors:** Yuya Sasajima, Makoto Miyata

**Affiliations:** ^1^Department of Biology, Graduate School of Science, Osaka City University, Osaka, Japan; ^2^The OCU Advanced Research Institute for Natural Science and Technology (OCARINA), Osaka City University, Osaka, Japan

**Keywords:** helix, MreB, fibril protein, SAH nucleosidase, electron microscopy, cooperativity, kink, evolution

## Abstract

*Spiroplasma* are helical bacteria that lack a peptidoglycan layer. They are widespread globally as parasites of arthropods and plants. Their infectious processes and survival are most likely supported by their unique swimming system, which is unrelated to well-known bacterial motility systems such as flagella and pili. *Spiroplasma* swims by switching the left- and right-handed helical cell body alternately from the cell front. The kinks generated by the helicity shift travel down along the cell axis and rotate the cell body posterior to the kink position like a screw, pushing the water backward and propelling the cell body forward. An internal structure called the “ribbon” has been focused to elucidate the mechanisms for the cell helicity formation and swimming. The ribbon is composed of *Spiroplasma*-specific fibril protein and a bacterial actin, MreB. Here, we propose a model for helicity-switching swimming focusing on the ribbon, in which MreBs generate a force like a bimetallic strip based on ATP energy and switch the handedness of helical fibril filaments. Cooperative changes of these filaments cause helicity to shift down the cell axis. Interestingly, unlike other motility systems, the fibril protein and *Spiroplasma* MreBs can be traced back to their ancestors. The fibril protein has evolved from methylthioadenosine/S-adenosylhomocysteine (MTA/SAH) nucleosidase, which is essential for growth, and MreBs, which function as a scaffold for peptidoglycan synthesis in walled bacteria.

## Introduction

*Spiroplasma* is a parasitic bacterium that infects arthropods and plants globally (Regassa and Gasparich, 2006; Harne et al., 2020b). Their interactions with hosts are mostly commensal but sometimes pathogenic, causing economical damage to different industries (Regassa and Gasparich, 2006). Interestingly, *Spiroplasma poulsonii* is known to disrupt the sex ratio of *Drosophila* species by killing males (Regassa and Gasparich, 2006; Harumoto and Lemaitre, 2018). Their successful survival may be supported by a unique swimming mechanism, which may be advantageous for translocation in the tissues of their hosts, because they do not stack due to high load to their appendages as do flagella and pili, which are widespread in bacterial motility (Miyata et al., 2020; Nakamura, 2020). *Spiroplasma* possesses helical cell morphology and swims in viscous media by switching handedness (Shaevitz et al., 2005; Wada and Netz, 2009).

The genus *Spiroplasma* belongs to the phylum Tenericutes, composed of the class Mollicutes, which evolved from the phylum Firmicutes represented by *Bacillus* and *Clostridium*. Mollicutes have some of the smallest genome sizes among all culturable organisms and lack a peptidoglycan (PG) layer, unlike other bacteria (Razin et al., 1998; Grosjean et al., 2014; Miyata et al., 2020). These unique characteristics were established during the evolutionary process of Mollicutes from Firmicutes. Many bacterial species, including *Escherichia coli* and *Bacillus subtilis*, can grow in the L-form, which does not synthesize the PG layer under stresses inhibiting peptidoglycan maintenance (Claessen and Errington, 2019). Mollicutes may have survived in the L-form due to their ability to escape the innate immune system of their hosts by halting the synthesis of PG, a major target of natural immune system of hosts (Claessen and Errington, 2019). During the evolution from a Firmicutes-like ancestor to extant Mollicutes, they established stable parasitism with the acquisition of adhesion ability, modulation of antigenic properties, and reduction in metabolic pathways. In the absence of the PG, the flagella-based motility common in Firmicutes was lost because the machinery is anchored to the PG layer (Miyata et al., 2020). Hence, Mollicutes may have evolved new motility systems because motility is beneficial for parasitic life. Interestingly, in addition to *Spiroplasma* swimming, Mollicutes have two types of unique gliding motilities, even though they are a small group, as discussed previously (Miyata and Hamaguchi, 2016a,b). The mechanism of *Spiroplasma* swimming has attracted many researchers in the fields of mycoplasmology, motility, and structural biology. Although the mechanism has been discussed in some aspects, a model for the whole image has not emerged (Kürner et al., 2005; Shaevitz et al., 2005; Trachtenberg et al., 2008; Wada and Netz, 2009; Roth et al., 2018; Harne et al., 2020a,b; Nakane et al., 2020). In this perspective review, we suggest a working model to explain the swimming mechanism and its evolution based on currently available information and ideas.

## Main Text

### Swimming Scheme

**FIGURE 1:**
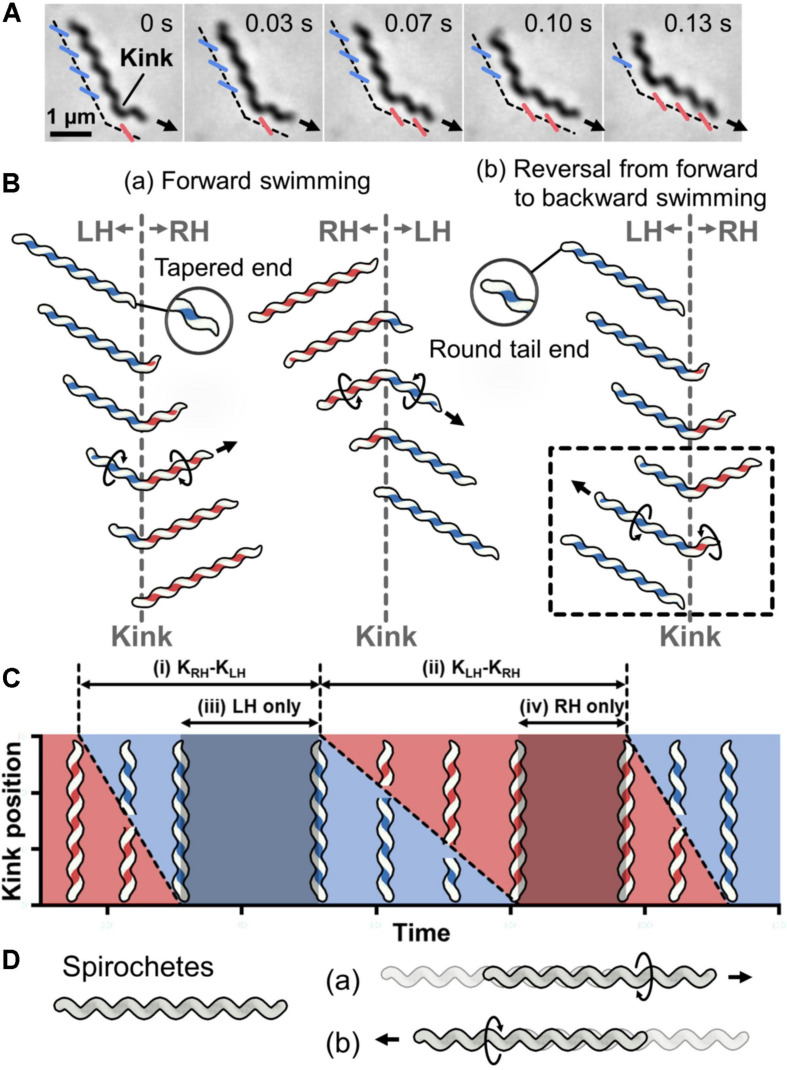
Helicity-switching swimming of *Spiroplasma*. **(A)** Phase-contrast microscopy of swimming cell. The blue and red lines indicate the left- and right-handedness (LH and RH) of the helical cell morphology, respectively. The swimming direction is indicated by the arrow. Time is shown in seconds at the upper right of each panel. **(B)** Swimming schematics. A kink is formed at the boundary of the different handedness. Counter-rotational torque is generated backward and forward of the kink position as shown by circular arrows. (a) The cell basically swims in the direction of the tapered end. (b) Sometimes cells reverse the direction of kink traveling, resulting in swimming reversal. The reversal kink traveling is presented in the dashed box. **(C)** Schematic diagram of kink position as it travels along the whole cell length during swimming. The time when the cell helix keeps LH and RH is colored blue and red, respectively. The intervals of the kink productions are indicated by (i) and (ii). The time when the cell keeps the body as LH and RH is marked as (iii) and (iv), respectively. This is a schematic presentation of previous data (Nakane et al., 2020). **(D)** Schematic for Spirochetes swimming. The helical cell has the same handedness throughout the whole cell length. The cell swims forward by rotating the cell helix in one direction (a) or backward after switching the rotational direction (b).

*Spiroplasma* swimming has been analyzed for three species, *Spiroplasma melliferum*, *Spiroplasma citri*, and *Spiroplasma eriocheiris* (Shaevitz et al., 2005; Wada and Netz, 2009; Liu et al., 2017; Roth et al., 2018; Harne et al., 2020a; Nakane et al., 2020; Sasajima et al., 2021). They are characterized as a helical cell 2–10-μm long with a tapered end (Figures 1A,B) (Supplementary Video 1). They swim up to 5 μm/s in viscous media by dynamically switching their handedness. The cells have different handedness simultaneously localizing along the cell axis, reversing the handedness from the tapered end, making a kink at the boundary of the axis (Shaevitz et al., 2005; Wada and Netz, 2009; Nakane et al., 2020). When the kink travels along the cell axis, the helical structure on one side of the kink rotates in one direction, and the other side rotates in the opposite direction to counteract the torque (Figure 1B). These two directional rotations caused by the helicity shift generate a propulsion force in one direction. When the environmental viscosity increases, the swimming speed increases while maintaining the traveling speed of the kink, suggesting that the force is transmitted from helix rotation to water *via* friction (Shaevitz et al., 2005). In more detail, the cell pushes water backward by rotating the backside cell body from the kink position like a propeller, and the cell progresses like a corkscrew (Figure 1B) (Wada and Netz, 2009). When the cell swims in one direction, the distributions of helix handedness, generation, and traveling of the kink in a cell can be presented as shown in Figure 1C (Nakane et al., 2020). Note that the cell architecture is composed of proteins, and that the structures and behaviors have intrinsic chirality not in mirror images (Sasajima et al., 2021).

No other motility system is driven by switching the helical handedness of a cell (Miyata et al., 2020; Nakamura, 2020). Although Spirochetes, which are a phylum of Gram-negative bacteria, are also known as helical swimming bacteria, their swimming system is completely different from that of *Spiroplasma* (Figure 1D) (Liu et al., 2009; Charon et al., 2012; Nakamura, 2020). Spirochetes rotate and propel the cell body by rotating their flagella that are aligned along the cell axis in the periplasmic space.

### Cell Architecture in Swimming

**FIGURE 2:**
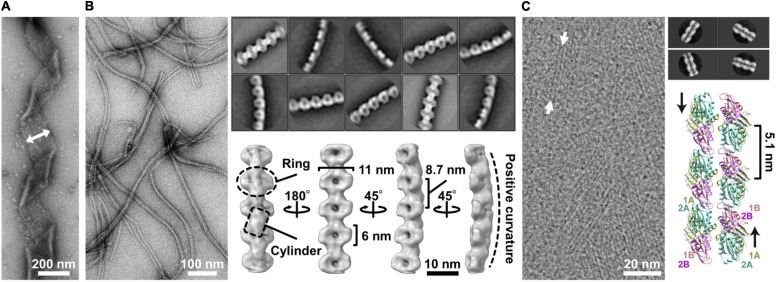
Ribbon components. **(A)** Negative staining electron microscopy (EM) of the ribbon. The ribbon width is 150 nm long and is indicated by the two-headed arrow. **(B)** Structure of the fibril filament. Negative staining EM of the isolated fibril filament (left). Two-dimensional class averages derived from the electron micrographs of fibril filaments (right upper). The three-dimensional structure of the fibril filament (right lower). A ring and a cylinder are marked by an oval and a box, respectively. The filament has 8.7-nm periodicity along the axis and positive curvature as marked by a broken line. **(C)** CryoEM image of *Spiroplasma citri* SMreB5 filament (left). Averaged image of the double-stranded MreB filament (right upper). A model of double protofilament of *S. citri* MreB5 generated by superposing the structure of *S. citri* SMreB5 (PDB ID: 7BVY) on *Caulobacter crescentus* MreB (4CZE). This panel was reorganized from a previous article (Harne et al., 2020a) with permission.

**FIGURE 3:**
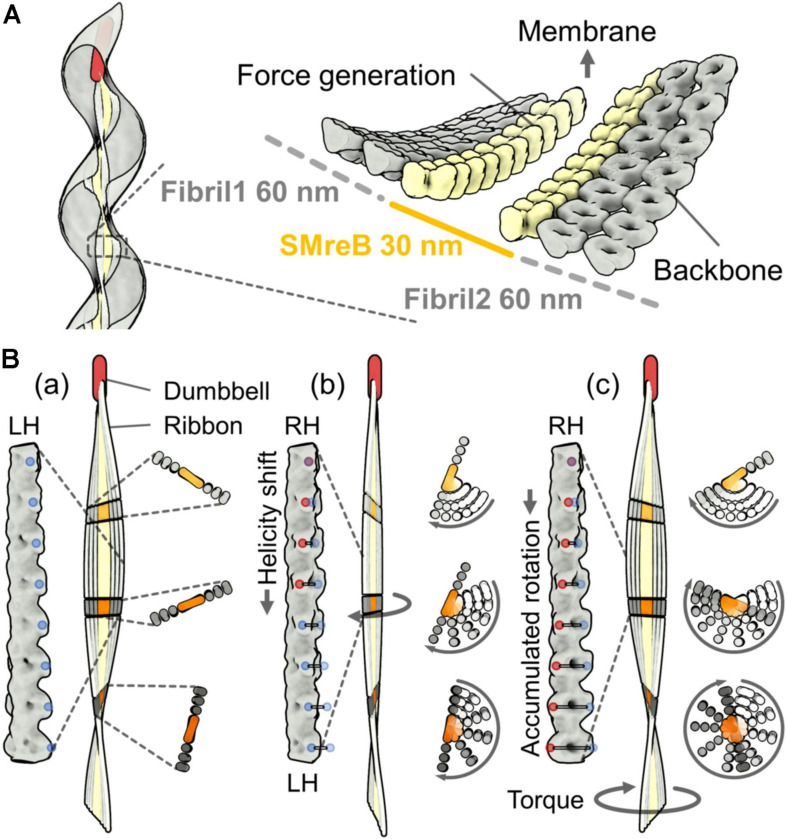
A working model for the helicity shift showing the propulsion of the cell body by pushing the water backward. **(A)** Left: Ribbon alignment along the innermost line of the helical cell body. Right: A model for ribbon composed of SMreB and fibril filaments, suggested from cryoEM (Kürner et al., 2005). The fibril protein is aligned in the outer zones that are each 60 nm wide. SMreB molecules are aligned in the inner zone that is 30 nm wide. **(B)** Ribbon rotation caused by accumulated twists. A left-handed ribbon (a), a transition state from left-handedness (LH) to right-handedness (RH) (b), and the resulting right-handed ribbon (c) are presented. The magnified images of the fibril filament in the ribbon, the whole ribbon, and the small stack of the ribbon are shown on the left, center, and right panels, respectively, for the three conformations (a), (b), and (c). The ribbon is fixed at the cell front by a dumbbell structure. The rotation of the small stack from conformation (a) is shown by traces with different transparencies in (b) and (c). The twists of the fibril filaments accumulate with the ribbon rotation. The accumulated twists push the water backward and generate a propulsion force. LH and RH points are marked by blue and red dots, respectively. The traces of the colored dots from the original positions in (a) are shown as horizontal lines in (b) and (c).

The characteristic helical cell shape of *Spiroplasma* is maintained by an internal ribbon approximately 150 nm wide, which runs the whole cell length (Figure 2A) (Kürner et al., 2005; Trachtenberg et al., 2008; Liu et al., 2017; Sasajima et al., 2021). The ribbon, composed of sheets of cytoskeletal filaments, is aligned along the innermost line of the helical cell structure. It can be divided into the central zone, 30 nm wide, and outer zones based on electron microscopy (EM) (Kürner et al., 2005). In the outer zone, “fibril” filaments are suggested to be aligned horizontally with 10-nm periodicity (Kürner et al., 2005). Fibril protein is specific to the *Spiroplasma* genus and has been the focus of research since 1980 (Figure 2B) (Townsend et al., 1980; Trachtenberg et al., 2008, 2014). Recently, the critical role of fibril filaments has been suggested based on its structure determination using EM (Sasajima et al., 2021). The structure was consistent with previous works but did not suggest contraction and extension, as expected previously (Kürner et al., 2005; Cohen-Krausz et al., 2011). Instead, it suggested roles as the determinant of cell helicity. The fibril filament is composed of oval rings with dimensions of 11 and 6 nm, a cylindrical connecting part, and aligned with an 8.7-nm unit length (Figure 2B) (Sasajima et al., 2021). The units do not disassemble or change its length; however, each unit is twisted either side relative to adjacent ones. The side view of the fibril filament showed that the cylindrical part formed a positive curvature (Figure 2B). In a cell, the fibril filaments are aligned beneath the membrane with this curvature, forming parts of the ribbon (Figure 3A) (Kürner et al., 2005; Sasajima et al., 2021). The isolated fibril filaments showed half pitches around 350 nm similar to those of cells, suggesting that the cell helicity is determined by fibril filaments. As the helicity shift for swimming travels along the cell axis, the shift in fibril filaments likely transmits to the next subunit probably through the strong cooperativity of fibril filaments along the ribbon axis (Nakane et al., 2020), accumulating their twists. The twists rotate the backside of the shift point, resulting in pushing the water backward (Figure 3B). In our current model, fibril filaments determine the cell helicity and its shift. Then, what generates the force for helicity shift of fibril sheet? We focus on another major component, MreB.

Filaments of *Spiroplasma* MreB, a member of the actin superfamily, were suggested to associate laterally to the fibril sheet at two different positions by two independent studies (Kürner et al., 2005; Trachtenberg et al., 2008). Here, we focus on the MreB filaments forming the inner zone of the ribbon, 30 nm wide (Figure 3A) (Kürner et al., 2005). MreB, a widely conserved protein in rod-shaped bacteria, polymerizes into short antiparallel double-stranded filaments and binds to the cell membrane (Shi et al., 2018). Although walled bacteria have only one type of MreB, all *Spiroplasma* species have five classes of MreB (Ku et al., 2014; Harne et al., 2020a; Takahashi et al., 2020). Here, we refer to *Spiroplasma* MreB as SMreB because they are phylogenetically distant from those conserved in walled bacteria. In a previous study using a *S. citri* mutant with a swimming defect, SMreB5 was shown to be essential for helicity formation and swimming (Harne et al., 2020a; Pande et al., 2021). It forms antiparallel double-stranded filaments and binds to both the membrane and fibril protein (Figure 2C).

The ribbon binds to a rod structure named “dumbbell,” which is about 240 nm long, at the tapered end of a cell (Figure 3A) (Liu et al., 2017). The dumbbell structure that forms the tapered end may contribute to swimming directionality. In contrast to the two types of gliding motilities in class Mollicutes (Miyata, 2007; Miyata and Nakane, 2013), *Spiroplasma* shows obvious chemotactic behavior toward amino acids and sugars (Liu et al., 2017). They change the swimming direction by reversing the direction of kink traveling as well as by changing the reversal frequency (Figure 1B). Interestingly, the genes of the two-component regulatory system, involved in all chemotaxis systems of other bacteria, were not found in the *Spiroplasma* genomes. This implies that *Spiroplasma* has a completely new chemotaxis system or that it has a two-component regulatory system distantly related to the common type. The dumbbell may be responsible for both cell polarity and control of the swimming direction.

### Possibility for Force Generation Mechanism

Generally, bacterial motility is driven by energy from ATP or the membrane potential (Miyata et al., 2020). As Mollicutes have no respiratory pathway, the energy for cell activities is thought to be supplied through ATP, which is produced by metabolic pathways (Fraser et al., 1995; Yus et al., 2009). Therefore, ATP should be more efficient as an energy source for Mollicutes motilities. In fact, two types of gliding systems, found in *Mycoplasma mobile* and *Mycoplasma pneumoniae*, are based on ATP energy (Uenoyama and Miyata, 2005; Kinosita et al., 2014; Mizutani and Miyata, 2019). *Spiroplasma* swimming can be stopped by the addition of carbonyl cyanide 3-chlorophenylhydrazone (CCCP), a proton ionophore (Nakane et al., 2020), suggesting that the energy for swimming is supplied from membrane potential. However, CCCP may affect other than membrane potential because much higher concentration was required to stop the swimming. If *Spiroplasma* swimming is also driven by ATP energy as other Mollicutes motilities (Uenoyama and Miyata, 2005; Kinosita et al., 2014; Mizutani and Miyata, 2019), SMreBs are candidates for force generators. In fact, polymerization dynamics based on ATP energy are known for conventional MreBs and SMreBs (Nurse and Marians, 2013; Gayathri, 2017; Takahashi et al., 2021). The stability of SMreB filaments in swimming cells remains unclear. However, they are likely to retain some filaments, as supported by the binding ability of SMreB5 to fibril filaments and liposomes (Harne et al., 2020a; Pande et al., 2021).

**FIGURE 4:**
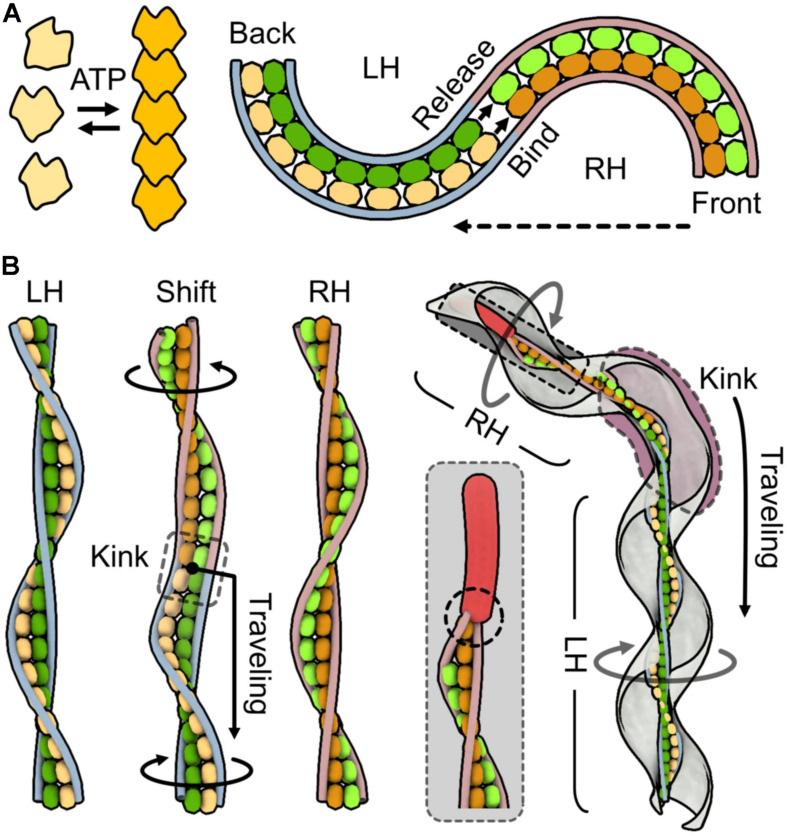
One working model for force generation and the transmission mechanism. SMreBs are presented as free and chained molecules. Two classes of SMreBs are colored yellow (light or dark) and green (light or dark). Fibril filaments are shown as blue [left-handedness (LH)] and red [right-handedness (RH)] strings without detailed structures for simplicity. Monomeric, polymerized, dissociated, and associated SMreB molecules are colored light yellow, dark yellow, light yellow or green, and dark yellow or green, respectively. **(A)** Bimetallic strip action of the ribbon suggested from characters of SMreB and fibril filaments. Left: Polymerization based on ATP energy is observed in some MreBs. Right: Helicity shift caused by length changes occurring differently in individual SMreB filaments. Left-handed ribbon shifts to right-handed from the front as shown by the broken arrow. The length changes in SMreB filaments related to polymerization dynamics of MreBs cause the helicity shift. Here, contraction is simply coupled to the association of molecules, but many other scenarios can be considered. **(B)** Three-dimensional presentation for helicity shift originated from bimetallic strip action of SMreB filaments. For simplicity, the ribbon is presented by the central paired SMreB filaments and the peripheral two fibril filaments. Left: LH, shifting (Shift), and RH ribbons are shown. In shifting, the yellow SMreB filament is switching from extended to contracted states, and the green filament is switching in the opposite way. Helicity shift propagates from upper to lower regions. A kink observed around the shifting point travels along the ribbon axis as shown by the black arrow, through the cooperativity of the structural changes. The upper and lower parts of the ribbon rotate relative to the switching point as indicated by black circular arrows. Right: Schematic presentation of a swimming cell. The dumbbell fixes filament movements at an end as marked by a broken circle, giving directionality to the ribbon. The kink travels down along the entire cell axis as shown by the black arrow, generating the counter-rotational torques in the front and back position of the kink. It also sometimes changes its traveling direction, showing the reversibility of the conformational changes.

Here, we attempt to explain the force generation mechanism. One simple idea is that MreB molecules assembled in the ribbon perform contraction and extension through its binding reactions, which are related to the polymerization dynamics. If the individual SMreB filaments are made by a uniform SMreB isoform, they can have specific timing for contraction and extension, resulting in a curve formation of a filament sheet. In more detail, an SMreB filament assembled in a ribbon can contract through subunit association and can extend through dissociation. This behavior is like a “bimetallic strip,” which is made of the combination of different metals and was previously widely used for switches in electricity circuits. If the change in the curvature of the SMreB filaments is large enough, it can cause a helicity shift (Figure 4A). Then, the helicity shift is refined in the structures of fibril filaments, which are specialized for the shift without change in length, and travels down along the ribbon and cell axes based on the axial cooperativity of the ribbon (Nakane et al., 2020). The idea that the helicity shift is caused by coordinated changes in filament lengths is common with a previously suggested model, although they assumed that fibril filaments aligned at outer zones change their lengths actively (Kürner et al., 2005). The dumbbell may have a role in triggering the helicity shift because the shift starts from the front (Figure 4B). The kink sometimes reverses the traveling direction to achieve chemotaxis, suggesting that cooperativity is not completely directed (Liu et al., 2017). Note that this working model is still an early version and will hopefully be refined by additional findings in the near future.

### Evolutional Origin of Swimming Mechanism

MreB, the ancestor of SMreB, plays the role of assigning “elongasome,” the complex for PG synthesis to appropriate positions, by detecting the cell membrane curvature (Shi et al., 2018). Although MreB belongs to the actin superfamily, it is currently not thought to control cell morphology through its filament structure. Therefore, the roles suggested for SMreBs appear far from those of conventional MreBs. The five classes of SMreB isoforms form a group at a position phylogenetically distant from the conventional MreB and closer to MreBH (Ku et al., 2014; Harne et al., 2020a; Takahashi et al., 2020). Each SMreB isoform is suggested to have different characteristics regarding ATP hydrolysis, filament formation, membrane binding, and interaction with other proteins (Harne et al., 2020a; Takahashi et al., 2020, 2021; Masson et al., 2021; Pande et al., 2021). It is possible that in the course of their evolution, the early stage of SMreBs separated into classes to obtain the bimetallic strip dynamics, which was suggested in Figure 4. *Haloplasma contractile*, belonging to class Mollicutes, is more related to the phylum Firmicutes than *Spiroplasma* is (Antunes et al., 2008). *H. contractile* cells have seven MreBs but no fibrils, whereas *Spiroplasma* has a single fibril protein (Ku et al., 2014; Takahashi et al., 2020). Interestingly, *H. contractile* switches between stretched straight and corkscrew-like contracted shapes. This suggests that *H. contractile* contracts by a mechanism related to the early version of *Spiroplasma* swimming (Antunes et al., 2008).

**FIGURE 5:**
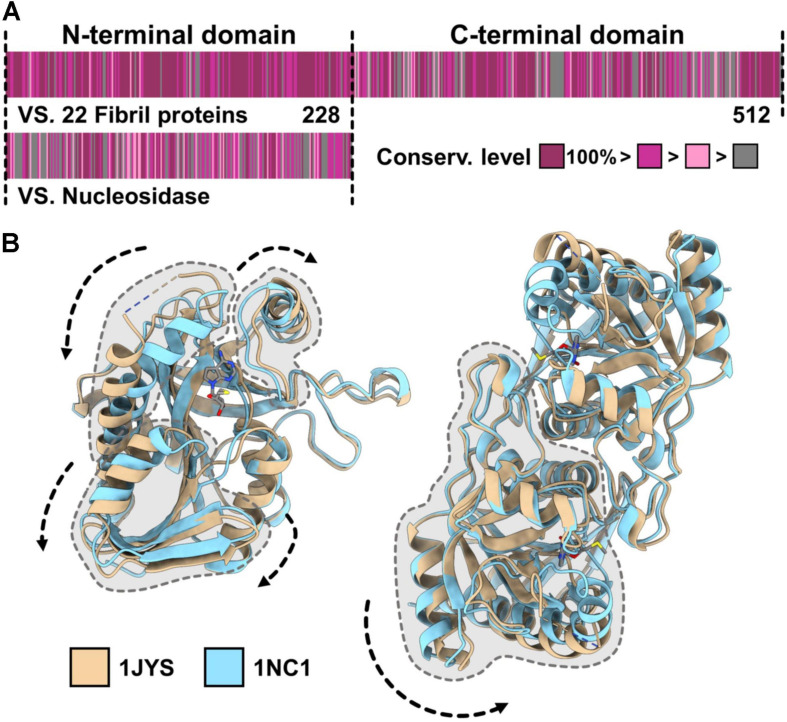
Evolutional origin of the fibril protein. **(A)** Amino acid sequence of the *Spiroplasma eriocheiris* fibril protein. The fibril protein, composed of 512 amino acids, can be divided into the N-terminal domain including 1–228 amino acids, which has homology to bacterial S-adenosylhomocysteine (SAH) nucleosidase, and the remaining fibril-specific C-terminal domain. The conservation level of the amino acid sequence among the fibril proteins from 23 *Spiroplasma* species (upper) and that against SAH nucleosidase from *Bacillus anthracis* (WP_098760943.1) are colored from gray (low) to purple (high) based on the Gonnet PAM 250 matrix (Larkin et al., 2007; Floden et al., 2016). **(B)** Conformational changes in the SAH nucleosidase. The two structures of SAH nucleosidase from *Escherichia coli* (PDB ID: 1JYS, 1NC1) are superposed. The conformational changes intra- (left) and inter-subunit (right) are indicated by dashed arrows.

The N-terminal domain of the fibril protein has approximately 20% amino acid sequence identity with an S-adenosylhomocysteine (SAH) nucleosidase, an enzyme that hydrolyzes S-adenosyl-L-homocysteine (Figure 5A). This protein is essential for bacterial growth because it recycles adenine and methionine by digesting methylthioadenosine (MTA) and SAH and also produces a quorum-sensing signal, autoinducer-2 (AI-2) (Parveen and Cornell, 2011). SAH nucleosidase is widespread in bacteria and is known to be a target for antibiotics. SAH nucleosidase forms a dimer by facing its N-terminal regions and undergoes conformational changes at the intra- and inter-subunit levels in the hydrolyzing reaction of the glycosyl bond (Figure 5B) (Lee et al., 2001, 2003). The structural changes in the fibril protein during swimming may originate from these conformational changes. The fibril protein showed conservation levels as high as 51%, with completely identical amino acids in all 22 *Spiroplasma* species (Figure 5A). This number is much higher than that of SAH nucleosidase, 10% in the 22 species, indicating that any mutation of the fibril protein is unacceptable in most amino acids if the fibril protein was not distributed through horizontal transfer.

## Perspective

Motility is thought to originate from the occasional transmission of large movements inside the cell, including DNA maintenance, ATP synthesis, and material transportation to the outside environment across the cell membrane (Miyata et al., 2020). However, this hypothesis has not been validated because few proteins involved in motility systems can be traced back to their ancestors. They were most likely refined rapidly during selection for efficient locomotion in their development. Swimming motility in *Spiroplasma* can be traced for its evolutionary origin, suggesting that the class Mollicutes is a young group of bacteria. If we trace this evolutionary process experimentally at the atomic level, it would provide a clue to clarify the principle underlying the evolution of both cell and motility.

## Author Contributions

YS made figures. Both authors wrote and organized the manuscript, contributed to the article, and approved the submitted version.

## Conflict of Interest

The authors declare that the research was conducted in the absence of any commercial or financial relationships that could be construed as a potential conflict of interest.

## Publisher’s Note

All claims expressed in this article are solely those of the authors and do not necessarily represent those of their affiliated organizations, or those of the publisher, the editors and the reviewers. Any product that may be evaluated in this article, or claim that may be made by its manufacturer, is not guaranteed or endorsed by the publisher.

## Supplementary Material

Supplementary Video 1 Phase-contrast microscopy of *Spiroplasma eriocheiris* in 0.6% methylcellulose. Real time. The image size was 37 μm × 23 μm.
